# Temporal trends in gender‐affirming hormone therapy initiation: evidence from whole‐of‐population Australian administrative data

**DOI:** 10.1111/imj.70194

**Published:** 2025-09-10

**Authors:** Karinna Saxby, Brendan J. Nolan

**Affiliations:** ^1^ Applied Economic and Social Research, Faculty of Business and Economics, Melbourne Institute University of Melbourne Melbourne Victoria Australia; ^2^ Trans Health Research Group, Medicine (Austin Health) University of Melbourne Melbourne Victoria Australia; ^3^ Department of Diabetes and Endocrinology Princess Alexandra Hospital Woolloongabba Queensland Australia; ^4^ Faculty of Medicine University of Queensland Brisbane Queensland Australia

**Keywords:** gender diverse, gender‐affirming hormone therapy, transgender

## Abstract

Using longitudinal national prescribing data, we estimated the number of transgender and gender‐diverse individuals initiating gender‐affirming hormone therapy (GAHT) from 2013 to 2024 in Australia. Between 2013 and 2024, 11 883 individuals initiated testosterone‐based GAHT and 20 358 initiated oestrogen‐based GAHT. Initiation rose from 1118 in 2013 to 5135 in 2024, with a growing share accessing testosterone‐based GAHT over time. The mean age at initiation also fell from 50 years in 2013 to 26 years in 2024. Our findings align with international trends, suggesting increasing demand among younger populations. This highlights the need for specialised, accessible gender‐affirming care in Australia and underscores the importance of improved data collection to inform health policy and clinical practice.

Transgender and gender‐diverse (TGD) people are those whose gender identity differs from their sex recorded at birth.^1^ Some TGD people seek gender‐affirming hormone therapy (GAHT) to align their physical characteristics with their gender identity, which is associated with improvements in mental health outcomes.^2^ In Australia, GAHT is publicly subsidised through the Pharmaceutical Benefits Scheme (PBS). Testosterone (parenteral or transdermal) is most commonly prescribed for individuals recorded female at birth, while oestradiol (oral or transdermal), typically combined with an anti‐androgen such as spironolactone or cyproterone acetate, is prescribed for individuals recorded male at birth.^3^, ^4^ Recent evidence suggests that the demand for GAHT is increasing,^5^ and in 2021–2022, approximately 18% of PBS‐subsidised testosterone prescriptions were estimated to be for gender affirmation.^6^ There is, however, currently no population‐level evidence on the number of people initiating GAHT in Australia, nor how this is changing over time. Our aim was to estimate the number of TGD Australians initiating PBS‐subsidised GAHT in Australia, and their average age at initiation, between 2013 and 2024.

The data for this analysis come from longitudinal PBS records spanning April 2012 to December 2024 (the most recent and complete data available at the time of writing) within the Person‐Level Integrated Data Asset.^7^ Following the previous literature,^6^ we identified TGD people accessing GAHT using information on gender markers and prescription records. Namely, TGD people accessing testosterone‐based GAHT (tGAHT) were classified as those who ever used testosterone (item codes 10378F, 08830R, 08619P,11740X, 10380H, 02115H, 10205D, 02114G) and had a current or previous female gender marker, and TGD people accessing oestrogen‐based GAHT (eGAHT) were classified as those who ever used oestradiol (item codes 13872D, 13980T, 14026F, 14642P, 14648Y, 14651D, 14652E, 14653F, 14660N, 1663M, 1664N, 8140K, 8274L, 8286D, 8311K, 8312L, 8761D, 8762E, 8763F, 8764G, 8765H, 8125P, 8485N, 8486P, 8126Q) and had a current or previous male gender marker. To increase confidence that we were observing initiation of GAHT, we excluded all individuals who were already receiving GAHT in 2012. Individuals prescribed both oestradiol and testosterone or those initiating younger than 15 years were also excluded.

The results for the study sample are presented in Figure 1. Between 2013 and 2024, 11 883 individuals initiated tGAHT and 20 358 initiated eGAHT. The number of people initiating GAHT increased steadily over time – from 1118 in 2013 to 5135 in 2024. From 2013 to 2024, most individuals initiating GAHT accessed eGAHT. In 2013, 93% of initiators accessed eGAHT, while only 7% accessed tGAHT. Over time, however, the proportion initiating tGAHT steadily increased, rising from 20% in 2016 to 41% in 2024. The mean age at initiation also fell, from 50 years in 2013 to 26 years in 2024. On average, eGAHT initiators were older than tGAHT initiators, but this difference reduced over time. Among tGAHT initiators, the decline in age was particularly steep between 2013 and 2018 but plateaued thereafter, with a consistent mean age of initiation between 24 and 25 years.

**Figure 1:**
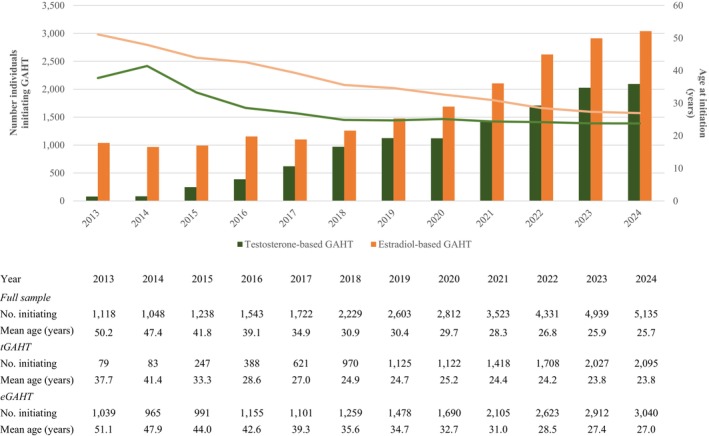
Individuals initiating PBS‐subsidised GAHT, Australia, 2013–2024. Age in years. Restricted to individuals initiating at 15 years and older. eGAHT, oestrogen‐based gender‐affirming hormone therapy; GAHT, gender‐affirming hormone therapy; PBS, Pharmaceutical Benefits Scheme; tGAHT, testosterone‐based gender‐affirming hormone therapy.

## Discussion

These results broadly align with another population‐level analysis in the Netherlands,^8^ which found that historically a higher share of TGD people accessed eGAHT, but that the number accessing tGAHT has been increasing. Recent Australian data also suggest that the increased access of tGAHT is driven by younger populations.^6^

There are several limitations to this analysis. First, as the PBS records only provide ‘M’ or ‘F’ gender markers, we cannot ascertain GAHT utilisation among non‐binary and other gender‐diverse Australians. Delays in the extraction of PBS records within Australian Person Level Integrated Data Asset (PLIDA), and associated claiming lags, also dictate that we will be missing some individuals initiating GAHT, particularly in more recent years. This analysis also excludes individuals accessing GAHT formulations that are not PBS subsidised. However, altogether these factors would lead us to underestimate the total population accessing GAHT. Finally, individuals in this sample may also include people with differences in sexual development or cisgender women with hypoactive sexual desire disorder. However, unsubsidised low‐dose testosterone formulations are generally used for this latter indication in Australia.^6^

Nevertheless, our findings demonstrate that the number of people initiating GAHT in Australia is increasing over time. This growing demand underscores the need for specialised, accessible care. To inform health policy and clinical practice, improved and standardised data collection on gender identity – such as in the upcoming Census^9^ – and an option for an additional formal indication for tGAHT on the PBS^6^ are essential.

The Office of Research Ethics and Integrity at the University of Melbourne has approved this study.

## Data Availability

Data are available upon request to the Australian Bureau of Statistics.
